# Semantic enhancement and cross-modal interaction fusion for sentiment analysis in social media

**DOI:** 10.1371/journal.pone.0321011

**Published:** 2025-04-28

**Authors:** Guangyu Mu, Ying Chen, Xiurong Li, Li Dai, Jiaxiu Dai

**Affiliations:** 1 School of Management Science and Information Engineering, Jilin University of Finance and Economics, Changchun, China; 2 Key Laboratory of Financial Technology of Jilin Province, Changchun, China; 3 Faculty of Information Technology, Beijing University of Technology, Beijing, China; University of Florida, UNITED STATES OF AMERICA

## Abstract

The rapid development of social media has significantly impacted sentiment analysis, essential for understanding public opinion and predicting social trends. However, modality fusion in sentiment analysis can introduce a lot of noise because of the differences in semantic representations among various modalities, ultimately impacting the accuracy of classification results. Thus, this paper presents a Semantic Enhancement and Cross-Modal Interaction Fusion (SECIF) model for sentiment analysis to address these issues. Firstly, BERT and ResNet extract feature representations from text and images. Secondly, the GMHA mechanism is proposed to aggregate important semantic information and mitigate the influence of noise. Then, an ICN module is created to capture complex contextual dependencies and enhance the capability of text feature representations. Finally, a cross-modal interaction fusion module is implemented. Text features are considered primary, and image features are auxiliary, enabling the profound integration of textual and visual features. The model's performance is optimized by combining cross-entropy and KL divergence losses. The experiments are conducted using a dataset collected from public opinion events on Sina Weibo. The results demonstrate that the proposed model outperforms comparison models. The SECIF model improves by 11.19%, 82.27%, and 4.83% over the average accuracy of the text-only, image-only, and multimodal models, respectively. The proposed SECIF model is compared with ten baseline models on the publicly available datasets. The experimental results show that the SECIF model improves accuracy by 4.70% and F1 score by 6.56%. Through multimodal sentiment analysis, governments can better understand public emotions and opinion trends, facilitating more targeted and effective management strategies.

## Introduction

The continuous development of information technology has made social media a vital part of people's daily lives [1,2]. Platforms like Sina Weibo, Twitter, and Facebook enable users to share their thoughts and experiences and play an essential role in forming and spreading social opinions [3]. Sentiment analysis helps analyze individual behavior and predict changes in public emotions, which can more objectively reflect the actual situation of online public opinion [4]. Implementing sensible regulatory policies can help governments curb the spread of false information and harmful content, mitigate the adverse effects of public opinion, create an orderly online environment, and maintain social stability [5,6]. Hence, it is significant to conduct sentiment analysis on social media.

In Natural Language Processing (NLP), sentiment analysis is critical. It focuses on extracting and analyzing subjective information from data to understand users' emotions, attitudes, and psychological tendencies [7]. With social media's rapid development, sentiment analysis has been extensively applied to different domains, such as tourism, medicine, and education. In the tourism industry, sentiment analysis can evaluate tourists' satisfaction and needs, improving service quality. In the medical field, it aids doctors in understanding patients' psychological states, enabling more effective treatment of mental issues like depression. In education, sentiment analysis helps teachers comprehend students' emotional changes, allowing them to adjust teaching strategies and enhance educational outcomes [8,9].

The development of sentiment analysis has progressed from traditional unimodal to multimodal methods [10]. Early sentiment analysis primarily relies on the text-based sentiment lexicon to identify emotional vocabulary within texts [11–18]. With the rise of machine learning and deep learning, sentiment analysis has achieved higher accuracy and efficiency, including multimodal data analysis such as images and sounds. In image sentiment analysis, researchers employ computer vision techniques to recognize facial expressions and body postures to confirm emotions [19–22]. In audio sentiment analysis, scholars utilize voice features such as tone, speed, and volume to determine speakers' emotions. For instance, a rising tone indicates happiness, while faster speech suggests anger [23,24]. However, unimodal features enable noise to be amplified during fusion, enhancing unimodal features as a critical issue. Furthermore, textual data significantly impacts sentiment analysis more than non-textual data. Thus, enhancing text features remains a primary research focus.

Due to the differences and inconsistencies in multimodal data, modality fusion has become an essential aspect of multimodal sentiment analysis [25]. Advances in deep learning have driven the development of multimodal fusion techniques, such as the Attention Mechanism, Long Short-Term Memory Network (LSTM), and Graph Neural Network (GNN), providing strong support for sentiment analysis [26,27,53]. By integrating various modalities, these methods are evident to improve the accuracy of sentiment analysis. However, multimodal fusion still needs to overcome many challenges. On the one hand, the heterogeneity and complexity of various modalities increase the difficulty of data processing. On the other hand, effectively removing noise and extracting useful features during fusion remains a problem that needs to be addressed.

This paper proposes the SECIF model to address these issues. The model comprises two core modules: a semantic enhancement module and a cross-modal interaction fusion module. The semantic enhancement module aims to improve feature extraction capabilities using the proposed GMHA mechanism and ICN module. The GMHA mechanism aggregates important semantic information and reduces interference from noise. The ICN module captures complex contextual dependencies and enhances the ability of text feature representations. The cross-modal interaction fusion module minimizes the differences and inconsistencies between different modalities. The efficiency and advantages of the SECIF model in social media sentiment analysis are validated using a self-scraped Sina Weibo public opinion dataset and two publicly available datasets.

The main contributions of this paper are as follows

Firstly, a novel sentiment analysis model called SECIF is established to bridge the semantic representations between text and images for more accurate results.
Secondly, the proposed GMHA mechanism aggregates important semantic information, reducing interference from noise.
Thirdly, the created ICN module captures complex contextual dependencies, enhancing the capability of text feature representations.
Finally, experiments are conducted using a self-scraped Sina Weibo public opinion dataset and two publicly available datasets to verify the accuracy and dependability of the model in social media sentiment analysis, further demonstrating the superiority of the SECIF model.

## Related work

This section provides a comprehensive review and discussion of existing literature on unimodal and multimodal sentiment analysis methods.

### Unimodal sentiment analysis

#### Text-based sentiment analysis.

Text sentiment analysis aims to extract emotional information from textual content through parsing and processing. It achieves a deep understanding and precise expression of sentiment. Text sentiment analysis can currently be classified into three primary approaches: lexicon-based, machine learning-based, and deep learning-based methods.

Lexicon-based methods depend on pre-constructed sentiment dictionaries to match the polarity of words in the text. It calculates the overall sentiment inclination through counting or weighted summation of every word in the content. Nguyen, Bermudez, and Yan et al. developed sentiment lexicons for Vietnamese, Spanish, and Tibetan to support sentiment analysis in different languages [12–14]. Similarly, Mu, Jiang, and Liu et al. created lexicons for finance, music, and photography to address the specific sentiment analysis needs within those fields [15–17]. Zhao et al. collected approximately 146 million Weibo texts to construct a large-scale sentiment lexicon. The lexicon contains about 100,000 words for sentiment analysis using simple text analysis methods [18]. However, sentiment lexicons often fail to cover all emotional expressions due to the continuous emergence of new words and the ambiguity of polysemous words.

Machine learning-based methods utilize labeled training data to build models for predicting text sentiment. Zhang et al. introduced a text sentiment analysis method using three-layer granularity, employing a three-branch decision region and support vector machine (SVM) for three-class classification [28]. Tang et al. integrated conditional random fields and dependency syntax rules to extract features for sentiment analysis [29]. Yang et al. utilized the Latent Dirichlet Allocation (LDA) topic model to extract eight environmental themes from a Weibo dataset and applied the eXtreme Gradient Boosting (XGBoost) ensemble model for sentiment analysis [30]. Zeng et al. applied LDA to extract topic distribution features from Weibo texts and employed the Adaptive Boosting (AdaBoost) ensemble classifier to develop the sentiment analysis model [31]. However, machine learning-based methods often rely on vast labeled data, are sensitive to noise, and struggle with handling long-distance dependencies.

Deep learning-based methods transform text into vector representations and build neural network models to capture context semantic information for predicting sentiment inclination. Deep neural network models are widely used in sentiment analysis because they effectively capture long-distance dependencies in text [32–34]. Jia et al. reconstructed weighted adjacency matrices for semantic and syntactic graphs, improving the accuracy of aspect-level sentiment analysis [54]. Lu et al. employed GNN to model intra- and inter-modal information to enhance dialogue emotion recognition [55]. Qian et al. used dual BERT models and a dynamic routing algorithm to capture and fuse contextual relationships [35]. The application of deep learning methods in sentiment analysis has become widespread, driving progress through emerging technologies.

#### Image-based sentiment analysis.

Image feature extraction plays a crucial role in computer vision. It aims to identify essential features within images or video frames and lays the foundation for subsequent image processing and analysis. Image sentiment analysis is divided into two main approaches: machine learning-based and deep learning-based methods.

Machine learning-based methods extract visual features from images, including shape, color, and texture, for training and recognizing emotions. Yanulevskaya et al. applied SVM to extract Wicce’s and Gabor’s features from images for emotion prediction [58]. Wang et al. employed Logistic Regression (LR) and SVM to predict the sentiments of social media images [19]. Gherkar et al. used SVM to analyze emotions in users' facial photos in restaurant reviews [20]. However, these methods can bridge the semantic representations between basic visual features and complex emotional semantics, reducing the accuracy of sentiment analysis.

Deep learning-based methods simulate the human neural network to extract emotional features in images, effectively addressing the semantic gap. Convolutional Neural Network (CNN) has gained widespread attention as the core method for image feature recognition. Recently, deep CNN architectures like VGG16, VGG19, Xception, ResNet, and ViT have achieved remarkable success in computer vision. Fan et al. integrated VGG19 and Xception models at the feature, intermediate, decision, and hybrid levels to analyze the visual sentiment of public opinion networks [21]. Wang et al. developed a model based on ResNet that combines CBAM with non-local modules for visual sentiment analysis [22]. Yang et al. integrated ResNet and Transformer to extract local and global image features [59]. Additionally, tools like OpenFace2.0 [36] and Facet [37] have been employed in the recognition of facial expressions in images, enhancing the accuracy and depth of image sentiment analysis [38].

### Multimodal sentiment analysis

In real-world learning and expression, humans rely on multiple senses. In sentiment analysis, it is essential to combine text, images, and other multimodal data [10,23,24,26,27,56,57]. Since the modal information is complementary and interrelated, integrating information from various modalities improves sentiment analysis precision, enabling a more thorough understanding and articulation of emotions [39].

Multimodal fusion is the key to sentiment analysis. It aims to extract and combine essential details from multiple modalities, reduce redundant noise information, and achieve effective interaction among modalities. Early scholars adopt feature fusion and decision fusion methods. Feature fusion enhances model expressiveness by encoding features from various modalities, and decision fusion improves model accuracy by integrating independent decision results from other modalities [40–43]. Several scholars have advanced the development of multimodal sentiment analysis methods by adopting different technical approaches. Zeng et al. used heterogeneous graphs to integrate knowledge and achieve feature fusion from multiple sources [53]. Yang et al. applied contrastive representation learning and contrastive feature decomposition to improve the representation of multimodal information [56]. Zeng et al. applied a dynamic routing network to capture the consistency and difference features by fusing visual and audio features with text modality as the center [57].

Recently, more researchers have used attention mechanisms for modality fusion. It effectively captures the relevance and importance of features across various modalities by assigning different weights to them, thereby enhancing multimodal fusion [44,45]. Hu et al. used the joint attention mechanism to identify significant regions consistent with text and images. They also employed the interactive attention mechanism to focus on feature interaction between different modalities and achieved an effective multimodal feature fusion [46]. Li et al. implemented six-modal information interaction through the cross-modal interaction mechanism to enhance single-modal features. They used the multi-head self-attention mechanism to calculate semantic relevance between original and enhanced features, improving the ability to recognize emotional features [47]. Luo et al. concentrated on learning common feature representations of modalities. They used the cross-attention mechanism to allow each modality to gain auxiliary information from the features of other modalities [48]. In the fusion module, scholars utilize the attention mechanism to weigh and connect the emotional semantic consistent features of various modalities, enhancing the expression ability of modalities and suppressing the negative impact of weak modalities.

## Methods

**Fig 1:**
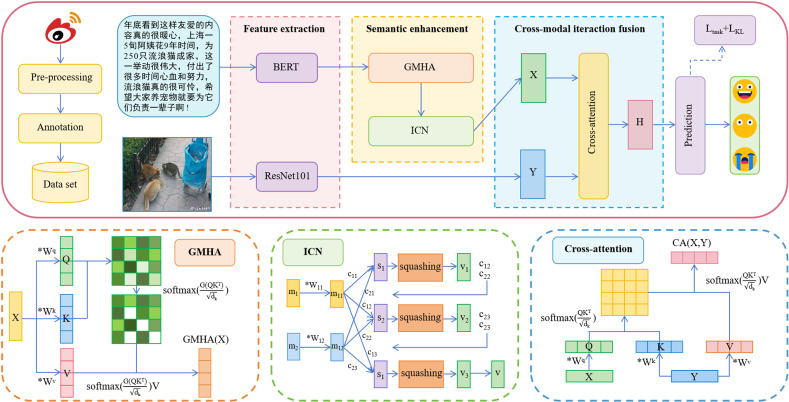
The overall framework of SECIF.

This section provides a detailed and comprehensive overview of the proposed SECIF model. Fig 1 displays the model's architecture. The model comprises four key components: feature extraction module, semantic enhancement module, cross-modal interaction fusion module, and sentiment prediction module. Each sample is divided into text and image modalities. The initial feature vectors are extracted for the text and images within the feature extraction layer. In the semantic enhancement layer, the textual feature vectors are processed by the innovative GMHA mechanism and the ICN module, which capture essential feature information and complex contextual dependencies, extending the capabilities of textual feature representations. The cross-modal interaction fusion module combines enhanced text and image features to achieve alignment and interaction across different modalities. Finally, we complete the sentiment analysis task.

### Feature extraction

#### Text feature representation.

Pre-trained language models are essential to extracting semantic features from text in NLP. Unlike Word2Vec, BERT addresses polysemy by utilizing contextual information and hierarchical learning. It acquires multi-level semantic features and provides rich feature options for downstream tasks. In multi-modal sentiment analysis, BERT is widely used to extract textual features. Specifically, given a text sequence *S*, tokens *[CLS]* and *[SEP]* are respectively inserted at the sequence's start and end of the sequence, denoted as *S =  [CLS, t1, t2,..., tn, SEP]*. The tokenized text sequence is passed through BERT’s embedding layer. The layer outputs the sum of token, segment, and positional embeddings. Equation (1) is used to calculate the text feature representation.


$$H_{T}=BERT\left(S\text{|}\theta_{T}^{BERT}\right)$$ (1)

Where *S* is the text sequence, $\theta_{T}^{\text{BERT}}$ denotes the parameters for extracting text features, and *H*_*T*_ indicates the feature representation of the text modality.

#### Image feature representation.

As a deep convolutional neural network, ResNet successfully solves the gradient vanishing and explosion problems in the traditional methods. The residual mechanism enables the network to learn the variation between the input and the desired output rather than the entire mapping. Hence, we employed the pre-trained ResNet101 model as the image encoder to extract high-level feature representations from images. Input images are resized to 768 × 768 and uniformly divided into 16 × 16 regions. The pre-trained ResNet101 model processes the original photos to obtain feature vectors. Equation (2) is used to calculate the image feature representation.


$$H_{I}=\text{ResNet}101\left(I\text{|}\theta_{I}^{\text{ResNet}}\right)$$ (2)

Where *I* indicate the original image, $\theta_{I}^{\text{ResNet}}$ denotes the image feature's extraction parameters, and *H*_*I*_ is the image modality's feature representation.

### Semantic enhancement

The proposed semantic enhancement module comprises the GMHA mechanism and the ICN module. The attention mechanism captures feature relationships and addresses information fragmentation and redundancy [34]. This paper proposes a GMHA mechanism by incorporating the Gather mechanism into the Multi-Head Attention (MHA) mechanism, effectively integrating information across different attention heads. The enhancement improves the model's complex data-handling ability and reduces interference noise. Capsule Network (CapsNet) is an emerging neural network architecture that captures hierarchical and spatial relationships of input data through the capsule layer structure. The approach allows them to represent complex syntactic and semantic relationships while reducing information loss from pooling operations. We create an ICN module that uses capsule vectors to capture complex contextual dependencies better, enhancing text feature representations' capability. Ultimately, this paper combines the GMHA mechanism and ICN module to improve feature extraction efficiency.

### Propose a GMHA mechanism

The MHA mechanism is widely applied in NLP, including machine translation, text classification, and language modeling. The input sequence is processed by multiple attention heads in the MHA mechanism. Each head generates an attention matrix that focuses on different aspects of the sequence. The attention matrices allow the model to capture various information, enabling simultaneous parallel processing. The MHA mechanism enhances the models' capacity to capture and aggregate semantic information within the input sequence, improving classification accuracy.

However, the existing MHA mechanism still needs help in feature quality, noise reduction, and data overfitting. This paper proposes a Gathered Multi-Head Attention (GMHA) mechanism to address these problems. The GMHA mechanism helps the MHA mechanism aggregate important feature information, reduce noise interference, and enhance effectiveness. In NLP, the Gather mechanism extracts critical information from text sequences by assigning weights based on word importance, filtering out the most representative elements. Specifically, the Gather mechanism assigns weights based on each word's importance in the text, sorts them, and selects the top *K* words with the highest weights as the critical information extraction results. Equation (3) is used to calculate the GMHA mechanism.


$$GMHA\left(G,Q,K,V\right)=GatheredMulti-HeadAttention\left(G,Q,K,V\right)=softmax\left(\frac{G\left(QK^{T}\right)}{\sqrt{d}}\right)V$$ (3)

Where *G* represents the Gather operation, and $Q=XW_{Q}^{t}$*,* $K=XW_{K}^{t}$, and $V=XW_{V}^{t}$ denote the projections of the input text sequence into query, key, and value spaces, respectively.

#### Create an ICN module.

In processing data, CNN fails to capture spatial relationships between low-level objects because the scalar values passed from one layer to the next cannot represent the relationships between low-level and high-level features. To address the limitations of traditional CNN, Hinton et al. proposed a new neural network model called CapsNet [49]. Unlike CNN, the vectors in CapsNet capture local features, which helps retain more feature information and spatial relationships. CapsNet applies dynamic routing algorithms to overcome the shortcomings of pooling layers and avoid losing feature information. It also captures complex syntactic and semantic relationships, which is advantageous for modeling relationships between words and phrases in a sentence.

However, CapsNet has limitations in updating the connection weights between capsules of dynamic routing. Inadequate adjustments of the weights can lead to training instability, ultimately impacting the model's performance. To further enhance the performance of the CapsNet, this paper creates an Improved Capsule Network (ICN) module. This module explicitly incorporates a modified squash function to improve numerical stability and ensure smooth, convergent training processes. Thus, the ICN module effectively captures and utilizes critical features to overcome traditional methods' disadvantages.

Firstly, a non-linear activation function transforms the fusion matrices into capsules. Then, the weight matrix *W*_*ij*_ learns the input features and computes the prediction vector *m*_*ij*_ for emotion capsule *j*, as shown in Equation (4).


$$m_{ij}=W_{ij}m_{i}$$ (4)

Where *m*_*i*_ represents the *i-th* feature capsule, indicating low-level emotion features. *m*_*ij*_ is the prediction vector for the emotion capsule *j*, and *W*_*ij*_ is the transformation matrix. The dynamic routing algorithm iteratively updates the weights between capsules to calculate the coupling coefficients to update *b*_*ij*_ values, as shown in Equation (5).


$$b_{ij}=b_{ij}+m_{ij}v_{j}$$ (5)

Where *v*_*j*_ denotes the vector output for emotion capsule *j*, and *b*_*ij*_ denotes the log-prior probability coupling between feature capsule *i* and sentiment capsule *j*. By updating *b*_*ij*_, *c*_*ij*_ is subsequently changed. Each capsule's vector length represents the probability of a specific emotion capsule, ranging from zero to one, as shown in Equation (6).


$$c_{ij}=softmax\left(b_{ij}\right)$$ (6)

Where *c*_*ij*_ denotes the weight assigned to capsule *j* by capsule *i*, and *b*_*ij*_ represents the raw coupling coefficients in the dynamic routing algorithm between capsule *i* and capsule *j*. Next, the sum of all *m*_*ij*_ with different weights generates *S*_*j*_. *S*_*j*_ denotes the *j-th* capsule in the sentiment capsule layer, as shown in Equation (7).


$$S_{j}=\sum c_{ij}m_{ij}$$ (7)

We modify the squash function to enhance numerical stability and ensure smooth and convergent training processes, as shown in Equation (8).


$$v_{j}=squash\left(S_{j}\right)=\frac{||S_{j}|{|}^{2}}{0.5+||S_{j}|{|}^{2}}\times \frac{S_{j}}{||S_{j}|{|}^{2}\sqrt{||S_{j}|{|}^{2}+T_{-}epsilion}}$$ (8)

Finally, the dynamic routing algorithm calculates the dot product of *m*_*ij*_ and *v*_*j*_ to update *b*_*ij*_. After completing the iterations, the routing process determines the final output, with the endpoint representing the correctly predicted capsule.

#### Cross-modal interaction fusion.

Increasing dynamic interactions between modalities is crucial in multi-modal sentiment analysis. Recent studies have shown that textual features contribute more significantly than non-textual to sentiment representation in multi-modal sentiment analysis [50–52,56]. Visual features may lead to discrepancies between sentiment categories and the authors' intentions. Scholars have adopted various fusion strategies and attention mechanisms to optimize the fusion of textual and visual features. However, these improvements still need to reduce the interference of visual features in sentiment representation. We utilize the cross-modal interaction fusion module built on the cross-attention mechanism to solve the issue. The method uses textual features as the primary focus, combining image features as an auxiliary component to achieve adaptive fusion and reduce potential visual feature interference. Additionally, the module allows feature representations from the modality to obtain auxiliary information from other modalities, promoting information exchange between modalities.

After obtaining the single modal sentiment features, we calculate the text and image features using the cross-attention mechanism. In the cross-attention mechanism, the inputs for Query, Key, and Value come from two sequences: *X* and *Y*. *X* inputs to Query, while *Y* inputs to Key and Value. The attention mechanism calculates the correlation between *X* and *Y* and multiplies the attention weights with Value to obtain cross-modal interaction features. The method enables adaptive fusion between modalities and reduces redundant information interference. Equation (9) is used to result in image-based text embedding representation.


$$H=Cross-Attention\left(X,Y\right)=softmax\left(\frac{\left(W^{\text{q}}X\right){\left(W^{\text{k}}Y\right)}^{\text{T}}}{\sqrt{d_{\text{k}}}}\right)\left(W^{\text{v}}Y\right)$$ (9)

*X* represents textual features, *Y* represents image features, *W* represents the linear transformation matrices of features, and *H* is the fused feature containing cross-modal interaction information.

By applying the cross-attention mechanism, we can leverage the image information to guide the text processing and achieve cross-modal information interaction and fusion. The method improves model performance and reduces overfitting risk.

### Sentiment analysis

After obtaining the final fused features *H*, we input them into a fully connected layer and then normalized them with softmax. Equation (10) is used to get the sentiment polarity *P*.


$$P=softmax\left(WH+b\right)$$ (10)

### Loss function

The Adam optimizer updates weights during the training phase in the model's classification task. We adopt the KL divergence loss. Equation (11) is used to calculate the KL divergence loss.


$$L_{KL}=\sum p\left(i\right)\cdot \log\left(\frac{p\left(i\right)}{q\left(i\right)}\right)$$ (11)

Where *p(i)* is the probability of the actual label being *i* and *q(i)* is the probability of the predicted label being *i*. The cross-entropy loss function evaluates the expected results and actual value discrepancy. Equation (12) is used to calculate the cross-entropy loss.


$$L_{task}=-\sum y_{i}\log\hat{y_{i}}$$ (12)

Where *y*_*i*_ denotes the actual label of sample *i*, and $\hat{y_{i}}$ is the probability of the predicted sample belonging to class *i*. Equation (13) is used to calculate the total objective loss.


$$Loss_{total}\text{=}L_{\text{KL}}+L_{\text{task}}$$ (13)

Where $L_{\text{KL}}$ is the KL divergence loss, $L_{\text{task}}$ is the cross-entropy loss, and $Loss_{total}$ is the total objective loss.

## Experiments and discussions

### Experimental setup

#### Dataset.

In this study, we scrape a series of public opinion events as our dataset on Sina Weibo, the largest online platform in China. The events include “Xu Shen Kindergarten,” “Datong Underage Bullying,” “Cultural Tourism Promotion,” “Demise of Four Major Fraud Families in Northern Myanmar,” and “Chongqing Siblings’ Death Case,” and so on, spanning from February 2023 to February 2024. A total of 11,165 instances containing both text and images are collected. We manually annotate the sentiment labels, categorizing them into positive, neutral, and negative sentiments. The final dataset is randomly divided into training and testing sets with a split ratio of 8:2. The distribution of the Weibo dataset is shown in Table 1.

**Table 1:** Distribution of the Weibo dataset.

Dataset	Positive	Neutral	Negative	Total
Training	2667	2864	3401	8932
Testing	665	715	853	2233

The data in this study is collected from publicly available sources, strictly following the terms and conditions of the source platform. The data analysis and processing are solely for academic research purposes. We ensure the legality of the collection and analysis method.

#### Implementation details.

Our experiments utilize a single RTX 4090 24GB GPU for training and testing. Model parameters are optimized with the Adam optimizer. The experiments are implemented in Python 3.12 and PyTorch 2.3.0, with CUDA 12.1 to support GPU-accelerated computation. The specific model parameters are listed in Table 2.

**Table 2:** Experimental parameters.

Parameter	Value
Text_maxlen	128
Batch_size	16
Learning_rate	1e-5
Dropout	0.1
Hidden_size	768
Attention_heads	12
Hidden_layers	12
Num_capsule	10
Dim_capsule	16
Routings	4
T_epsilion	1e-7
Epoch	10

#### Evaluation metrics.

To evaluate the effectiveness of the proposed model, we select several metrics, including accuracy, precision, recall, and F1-score. Equations (14)–(17) are used to calculate the evaluation metrics.


$$Acc=\frac{TP+TN}{TP+FP+TN+FN}$$ (14)

$$Pre=\frac{TP}{TP+FP}$$ (15)

$$Rec=\frac{TP}{TP+FN}$$ (16)

$$F1=\frac{2TP}{2TP+FP+FN}$$ (17)

*TP* denotes true positives, *TN* denotes true negatives, *FP* denotes false positives, and *FN* denotes false negatives. All evaluation metrics range from zero to one, with higher values reflecting better model performance.

### Experiment analysis

To evaluate the effectiveness of the proposed SECIF model, this section conducts a comparative experiment with different modalities, including text-only, image-only, and text-image bimodal modalities. Here is a brief introduction to the information on the models.

#### Text-only models.

1. Single models

**Sentiment Lexicon** A predefined sentiment lexicon is used to evaluate sentiment tendencies by matching words in the text.
**SVM:** Construct one or more hyperplanes in high-dimensional space to classify data effectively.
**BiLSTM:** Enhance the capture of contextual dependencies by considering both forward and backward context information in text data.
**BERT:** Utilize extensive pre-training on text data to capture complex contextual relationships through the Transformer structure, achieving deep semantic understanding.

2. Hybrid models

**BM:** Based on the BERT model, combined with multi-head attention to capture complex contextual relationships in text.
**BGM:** Extend BM by introducing the GMHA, enhancing the aggregation of textual information.
**BGMIC1**: Integrate BGM with an original CapsNet.
**BGMIC2** Similar to BGMIC1, but with the squash of capsule coefficient set to 0.5
**BGMIC3**: Integrate BGM and the ICN module

#### Image-only models.

**CNN:** Extract low- to high-level features from images through successive convolution operations.
**VGG16:** A 16-layer CNN with small 3x3 filters is used for image classification.
**ResNet50:** A deep residual network with 50 layers is used for image classification.
**ResNet101:** An extended version of ResNet50 with 101 layers, providing a more profound residual network architecture.

#### Text and image models.

**ElemAdd:** Combine text features from BGMIC3 and image features from ResNet101 through element-wise addition.
**Concat:** Concatenate text features from BGMIC3 and image features from ResNet101 features.
**ElemMul:** Combine text features from BGMIC3 and image features from ResNet101 through element-wise multiplication.
**BGMR:** Integrate text features from BGM with image features from ResNet101 using the cross-modal interaction attention mechanism. Text features serve as queries, and image features as keys and values, allowing interactive fusion of both modalities.
**BGMICR1:** Integrate text features from BGMIC1 with image features from ResNet101 using a cross-modal interaction attention mechanism.
**BGMICR2:** Text features from BGMIC2 with image features from ResNet101 are integrated using a cross-modal interaction attention mechanism.
**BGMICR3(SECIF)**: Text features from BGMIC3 with image features from ResNet101 are integrated using a cross-modal interaction attention mechanism, as proposed in this study.

In the single-text hybrid model, the attention mechanism, the Gather mechanism, and an improved capsule network are introduced in this paper, respectively. For further data enhancement, we fuse the image information and compare the performance of different text-image fusion models. Table 3 presents the experimental results of various models on the Weibo dataset.

**Table 3:** Experiment Results on the Weibo dataset.

Modality	Type	Model	Acc(%)	Pre(%)	Rec(%)	F1(%)
Text-only	Single models	Lexicon	67.94	77.05	67.94	67.16
		SVM	74.56	82.49	74.56	74.46
		BiLSTM	77.74	85.40	77.74	77.86
		BERT	84.19	84.65	84.30	84.18
	Hybrid models	BM	84.37	84.62	84.79	84.39
		BGM	85.71	84.65	85.91	85.73
		BGMIC1	86.12	84.62	85.93	86.17
		BGMIC2	86.74	84.67	86.90	86.67
		BGMIC3	88.09	88.40	88.00	88.12
Image-only	Single models	CNN	43.53	43.43	43.53	41.78
		VGG16	50.34	53.21	50.34	46.06
		ResNet50	52.44	52.53	52.44	50.63
		ResNet101	53.02	54.09	53.02	50.79
Text and image	Hybrid models	ElemAdd	81.96	81.84	81.89	81.85
		Concat	82.79	83.17	82.58	82.76
		ElemMul	87.33	87.62	87.04	87.16
		BGMR	88.90	88.95	88.79	88.86
		BGMICR1	89.21	89.15	89.01	89.11
		BGMICR2	89.83	89.73	89.84	89.73
		**BGMICR3(SECIF)**	**90.86**	**91.12**	**90.81**	**90.91**

Firstly, in the single text models experiment, SVM achieves a 9.74% higher accuracy than the traditional sentiment lexicon, demonstrating the advantages of machine learning in handling new vocabulary and polysemy. Additionally, the average accuracy of BiLSTM and BERT shows an improvement of 8.58% and 19.16% over SVM and sentiment lexicon, respectively. Deep learning models can better comprehend complex contextual information through bidirectional encoding techniques. Among the models, BERT performs the best, achieving an accuracy of 84.19% by leveraging rich linguistic features from large-scale corpora.

Secondly, in the hybrid text models experiment, the hybrid text methods are superior to single text methods. BM achieves a 0.21% higher accuracy than BERT, demonstrating that the multi-head attention mechanism can capture and integrate diverse semantic information from the text. The accuracy of BGM improves by 1.58% over BM. BGM achieves a 1.8% improvement over BERT, indicating that the proposed GMHA mechanism more effectively aggregates critical details within the text. The average accuracy of BGMIC1, BGMIC2, and BGMIC3 reaches 86.98%, reflecting a 1.48% improvement over BGM. This result demonstrates the capsule network can better understand semantic information and improve feature extraction efficiency. BGMIC3 achieves a 2.77% higher accuracy than BGM, further demonstrating the proposed ICN module's effectiveness in capturing complex contextual dependencies and enhancing the capability of text feature representations. Additionally, BGMIC3’s accuracy improves by 4.63% over BERT, showing the advantage of the semantic enhancement module in enhancing contextual feature extraction, leading to better classification performance.

Thirdly, in the image-only models experiment. VGG16’s accuracy improves by 15.64% over the traditional CNN, showing that increased convolutional layers enhance classification performance. The average accuracy of ResNet50 and ReaNet101 achieves a 4.74% improvement over VGG16, demonstrating that introducing residual blocks enhances image classification. ResNet101 achieves a 1.10% higher accuracy than ResNet50, indicating the increased number of residual blocks continues to improve classification performance.

In the comparative experiment between text and image models, the image models achieve an average accuracy of 49.83%. The text models achieve an average accuracy exceeding 80%, suggesting that text typically contains richer semantic information and emotional features, making it more suitable for analyzing sentiment on social media. The experiment demonstrates the efficacy of the strategy employed in this study, where text serves as the primary feature and images are used as auxiliary features.

Finally, in the text-image bimodal models experiment, simple concatenation methods, such as ElemAdd and Concat, result in lower accuracy than text-only hybrid models due to interference from image noise. The accuracy of ElemMul is lower than that of BGMIC3, indicating that ElemMul is a simple fusion method that needs more effective integration of text-image interactions. The average accuracy of BGMICR1, BGMICR2, and BGMICR3 improves by 1.20% over BGMR, demonstrating the advantage of the Capsule Network in enhancing understanding of semantic information and feature extraction efficiency. BGMICR3 achieves a 2.20% higher accuracy than BGMR, indicating the efficacy of the proposed ICN module in capturing complex contextual dependencies and enhancing the capability of text feature representations. Additionally, the average accuracy of BGMICR1, BGMICR2, and BGMICR3 is 89.97%, representing an improvement of 2.13% and 69.69% over the best-performing text model BGMIC3 and image model ResNet101. This improvement highlights the significant advantage of the cross-modal interaction and fusion module in integrating text and image features.

**Fig 2:**
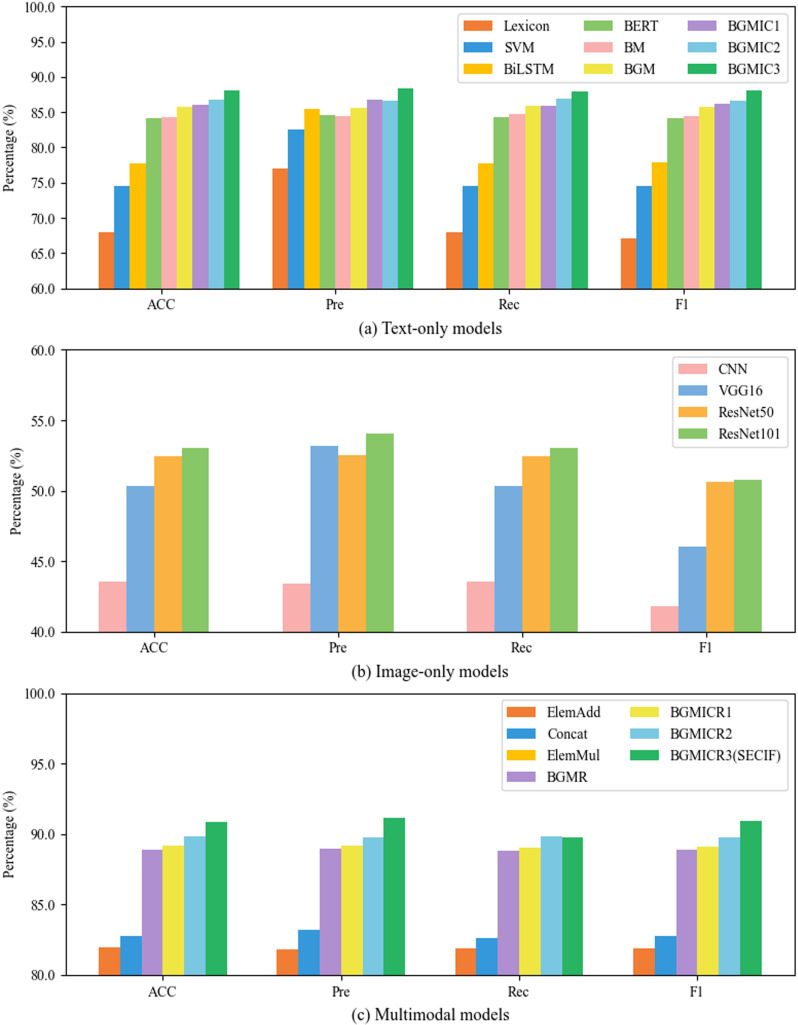
Comparison results on the Weibo dataset.

In general, the proposed SECIF (BGMICR3) model outperforms other models and effectively improves the accuracy of sentiment analysis predictions on social media. Fig 2 visualizes a more intuitive comparison of the performance differences among the models.

### Visualization analysis

**Fig 3:**
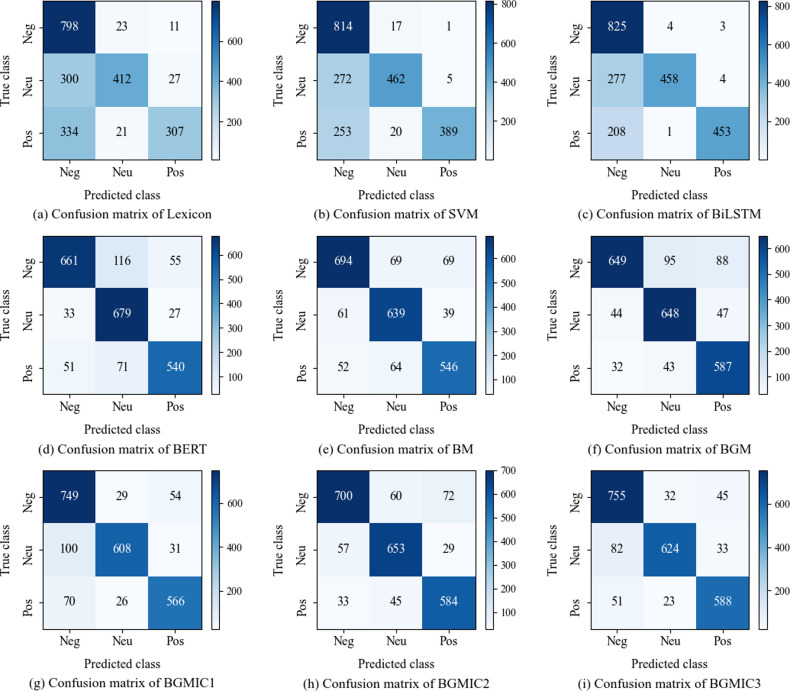
Confusion matrix of text-only models.

**Fig 4:**
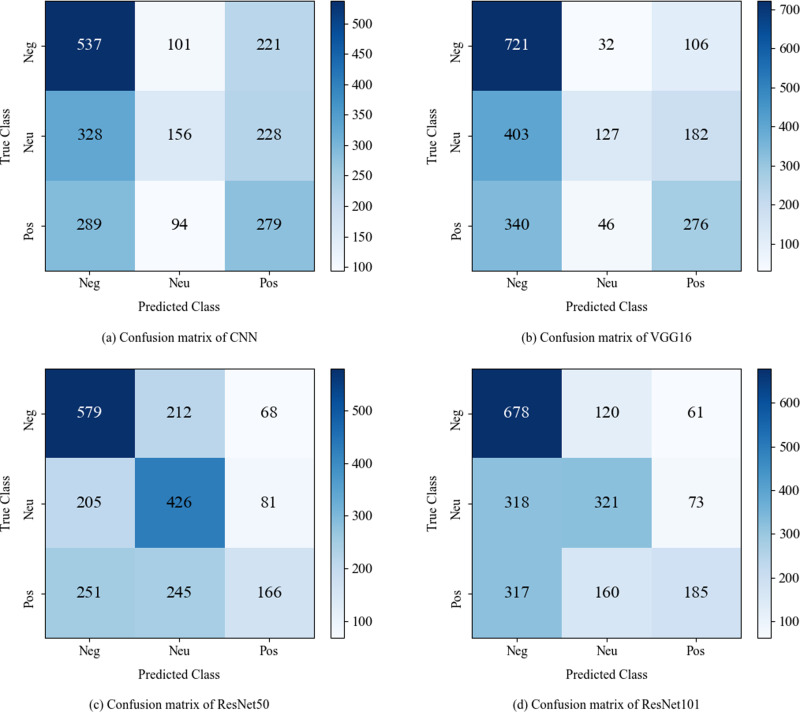
Confusion matrix of image-only models.

In this study, we utilize the confusion matrix to illustrate the classification performance of text-only and image-only models, as shown in Fig 3 and Fig 4. The visualization results provide intuitive representations of the models' performance in classifying positive, neutral, and negative sentiments, highlighting the models' strengths and weaknesses.

### Confusion matrix of text-only models

Fig 3 shows the classification visualization of text-only models. In the single text models experiment, the sentiment lexicon, SVM, and Bi-directional Long Short-Term Memory (BiLSTM) perform better at classifying negative sentiment but struggle with neutral and positive sentiments. The result indicates a higher sensitivity to negative sentiment in social media under the balanced data condition. In contrast, BERT shows outstanding performance across positive, neutral, and negative sentiment classifications, illustrating the superior ability to capture contextual information and handle complex sentiment information within the text.

In the hybrid text models experiment, BM outperforms BERT in classifying negative and positive sentiments, significantly reducing misclassifications across categories. The improvement suggests that the multi-head attention mechanism effectively captures semantic information from the text. Furthermore, BGM increases the number of correctly classified samples compared to BM, particularly in neutral and positive sentiment classifications, indicating the advantages of the GMHA mechanism in information aggregation. Compared to BERT, BGMIC1, BGMIC2, and BGMIC3 significantly improve classifying positive and negative sentiments. Among these models, BGMIC3 achieves the highest accuracy in classifying positive sentiment by introducing the ICN module, leading to the best overall classification performance. This experiment further validates the effectiveness of the proposed semantic enhancement module.

### Confusion matrix of image-only models

Fig 4 presents a visualization of the classification results of image-only models. The CNN model performs well in classifying negative sentiment but struggles with neutral sentiment. As the convolution layers increase, VGG16 improves negative sentiment classification compared to CNN. With the introduction of residual blocks, ResNet50 and ResNet101 enhance the accuracy of neutral sentiment classification and effectively reduce errors in negative sentiment classification. ResNet101, in particular, excels in neutral sentiment classification and further improves accuracy in identifying negative sentiment, demonstrating greater adaptability in sentiment analysis tasks.

### Loss rate analysis

**Fig 5:**
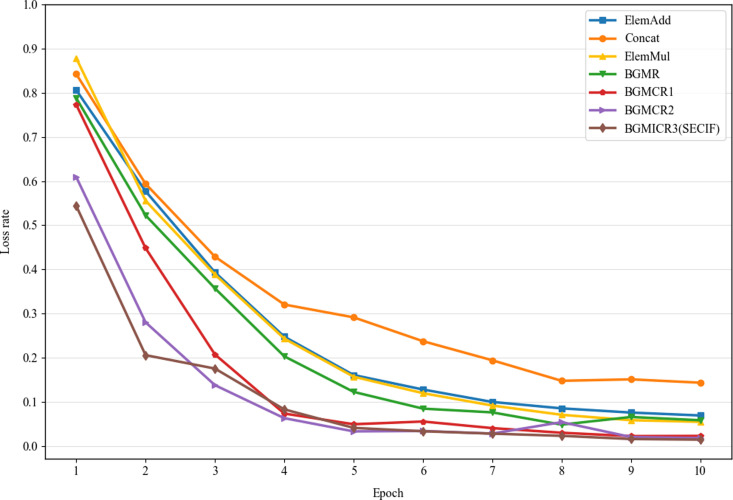
The loss rate of multimodal fusion models.

This study conducts a comparative analysis to evaluate the performance of various multimodal feature fusion strategies. Fig 5 shows the loss rate trends of the multimodal fusion models, highlighting differences in convergence speed and final performance.

Concat initially starts with a lower loss rate but experiences a slower decline, ultimately reaching the highest loss rate among the models, indicating Concat has a weaker aggregation capability in feature fusion. ElemMul shows a high initial loss rate but converges faster and achieves a lower loss rate than ElemAdd and Concat, indicating strong convergence ability. ElemAdd’s overall loss rate is similar to ElemMul’s, but it converges more slowly than ElemMul in the early stages. BGMR achieves a lower loss rate than ElemMul, indicating the importance of effective feature fusion in multimodal sentiment analysis. BGMICR1, BGMICR2, and BGMICR3 consistently maintain lower loss rates than BGMR, demonstrating the crucial role of the Capsule Network in rapid convergence. BGMICR1, BGMICR2, and BGMICR3 perform exceptionally well in sentiment analysis. Their loss rates decrease rapidly in the initial stages, stabilize by the fourth iteration, and reach the lowest overall loss rates, demonstrating the effectiveness of the cross-modal interaction fusion module. Additionally, BGMICR3 performs the best among all the fusion models, demonstrating the efficacy of the ICN module in capturing complex contextual dependencies and rapid convergence.

### Baseline models comparison experiment

#### Datasets.

We use two publicly available datasets in the experiment: MVSA-Single and MVSA-Multiple [60]. The MVSA datasets from Twitter consist of text-image pairs categorized as positive, neutral, and negative. The distributions of the MVSA datasets are shown in Table 4.

**Table 4:** Distributions of the MVSA-Single and MVSA-Multiple datasets.

Datasets	Positive	Neutral	Negative	Total
MVSA-Single	2683	470	1358	4511
MVSA-Multiple	11318	4408	1298	17024

#### Baseline models.

The following baseline models are employed for the comparative experiment to verify the superiority of the SECIF model in sentiment analysis.

**MultiSentiNet [**61**]:** Extract visual semantic information from images to identify key emotional words in the text and integrate features for prediction.
**HSAN [**62**]:** Use a hierarchical structure to represent tweet levels, extracting visual features from captions and employing a contextual attention mechanism for encoding.
**Co-MN-Hop6 [**63**]:** Iteratively capture and understand the relationship between text and images.
**MGNNS [**64**]:** Encode various modalities for hidden representations, utilize a multi-channel GNN for learning, and achieve multimodal fusion through an MHA.
**CLMLF [**65**]:** Use contrastive learning to align and fuse text and image features via a multi-layer fusion module.
**MVCN [**66**]:** Reduce redundant visual elements using a text-guided fusion module, maintain feature consistency with an emotion constraint, and address inconsistent labels with adaptive loss calibration.
**CiteNet [**67**]:** Employ contrastive learning for modality alignment and achieve multimodal fusion with an enhanced integration module.
**MIGSIE [**68**]:** Improve image-text interaction with a text-guided multi-channel module, use GNN for co-occurrence feature extraction, and integrate multimodal features through a multi-source representation module.
**DTN [**69**]**: Facilitate modality interaction using a deep cross-modal attention network and two-stage feature fusion with gating and attention to adjust weights dynamically.
**SRC-Model [**70**]**: Facilitate bidirectional image-text interaction using cross-modal attention, refine emotional representation with a gating module, and combine image and text features through an MHA.

#### Comparative experimental results.

In this study, a comparative analysis is conducted to evaluate the performance of the proposed model. The comparative experimental results are shown in Table 5.

**Table 5:** Experimental results of baseline models on the MVSA-Single and MVSA-Multiple datasets.

Model	MVSA-Single		MVSA-Multiple	
	ACC	F1	ACC	F1
MultiSentiNet	69.84	66.90	67.69	67.76
HSAN	69.88	66.90	67.96	67.76
Co-MN-Hop6	70.51	70.01	68.92	68.83
MGNNS	73.77	72.70	72.49	69.34
CLMLF	75.33	73.46	72.00	69.83
MVCN	76.06	74.55	72.07	70.01
CiteNet	76.09	74.67	72.89	70.35
MIGSIE	76.40	75.20	72.72	72.20
DTN	77.11	76.46	70.71	68.10
SRC-Model	78.32	76.79	72.81	70.13
**SECIF**	**79.10**	**78.74**	**73.09**	**72.79**

In the MVSA-Single dataset, the baseline models achieve a mean accuracy of 74.33% and an F1 score of 72.76%. The SECIF model improves accuracy by 6.42% and the F1 score by 8.21%. In the MVSA-Multiple dataset, the baseline models obtain a mean accuracy of 71.03% and an F1 score of 69.43%. The SECIF model demonstrates enhancements of 2.91% in accuracy and 4.84% in the F1 score. Experimental results show the significant performance of the SECIF model over the baseline models. The SECIF model introduces a semantic enhancement module for processing the initial features of the data, which effectively reduces the influence of noise and enhances the representation of text features. In addition, compared with the simple attention mechanism model, SECIF captures the relationship between text and image more accurately through the cross-modal interaction fusion module.

## Case study

**Fig 6:**
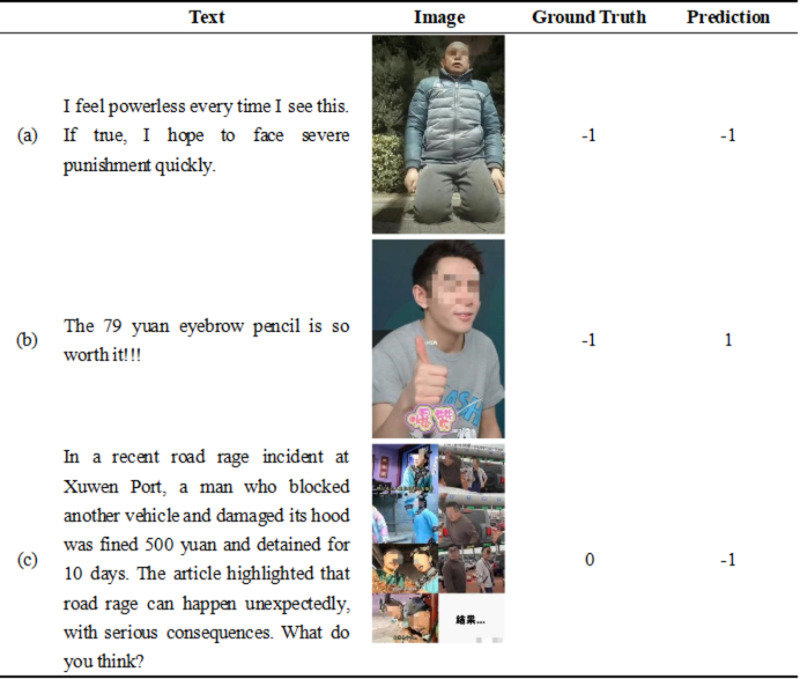
Examples of Ground Truth and Prediction.

This study examines the SECIF cases to determine the reasons for the errors and to suggest improvements. Fig 6 shows examples of correct and incorrect predictions.

In Fig 6(a), the text contains phrases such as “powerless” and “severe punishment quickly,” which express the blogger's negative sentiment. The male's serious and sorrowful facial expression also represents the same emotion. After combining the text and image, the model's prediction result matches the ground truth label, confirming the accuracy of the SECIF’s prediction. The phrase “so worth it” suggests a positive sentiment in Fig 6(b), further conveyed by the thumbs-up gesture and the smiling face. However, the real implication of the comment is that the eyebrow pencil is overpriced and does not match its actual value. The result reflects the sarcastic tone as well as the negativity of the blogger. The data lacks external knowledge, which restricts the understanding of the comments' context and leads to deviation in emotional analysis. In Fig 6(c), the text discusses a public opinion event's cause, outcome, and recommendations, suggesting a neutral sentiment. The spliced images fail to personalize the process during feature extraction. Some crucial details may be missing, which results in the loss of image information.

## Conclusions and future work

### Conclusions

Public opinion plays a crucial role in maintaining social stability in social media. As a result, it is necessary to build an accurate sentiment prediction model for public opinion. This study proposes a model based on Semantic Enhancement and Cross-Modal Interactive Fusion (SECIF), which significantly improves the accuracy of sentiment analysis on social media. The conclusions are as follows.

Firstly, the proposed GMHA mechanism in aggregating crucial semantic information is adequate. BGM achieves a 1.8% and 1.58% improvement over BERT and BM, respectively. These results show that the GMHA mechanism enables more accurate aggregation of vital semantic information, reducing interference from noise and improving sentiment classification accuracy.

Secondly, the proposed ICN module in capturing complex contextual dependencies is also essential. In the text-only models experiment, the average accuracy of BGMIC1, BGMIC2, and BGMIC3 achieves a 1.48% improvement over BGM. BGMIC3 achieves a 2.77% higher accuracy than BGM. Additionally, in the text-image bimodal models experiment, the average accuracy of BGMICR1, BGMICR2, and BGMICR3 improves by 1.20% over BGMR. BGMICR3 achieves a 2.20% higher accuracy than BGMR. These results show the effectiveness of the ICN module in handling long-distance dependencies and enhancing the capability of text feature representations.

Thirdly, the authenticity of social media data is compelling. The model is validated using a collected and annotated dataset of opinion images and texts from Sina Weibo, confirming the reliability of the experimental results and the feasibility of practical applications. The solid foundation for performance evaluation enhances the research outcomes' real-world significance and reference value.

Finally, our model demonstrates excellent performance on the two publicly available datasets. We compare the proposed model with ten baseline models. The experimental results indicate that the SECIF model improves accuracy by 4.70% and the F1 score by 6.56% compared to the baselines on the MVSA datasets.

In summary, the proposed semantic enhancement module significantly improves the model's capability to capture and understand sentiment expressions on social media. The proposed SECIF model elevates the performance of sentiment analysis, provides a powerful tool for public opinion management, and offers valuable insights for future research in this domain.

### Future work

In future work, we plan to conduct additional research to achieve even more accurate sentiment analysis in social media. On the one hand, the proposed model fails to understand the context of the comments in recognizing sarcastic and implied emotions. We will further introduce external knowledge to identify complex emotions. On the other hand, some vital details in the clipping operation may be missing, which will cause the loss of image information and affect sentiment analysis ability. We will improve image processing techniques by integrating image segmentation, spatial attention, and channel attention. Moreover, we will add more datasets to strengthen the model's ability to capture and understand user emotions on different platforms.
